# Cisplatin-induced epigenetic activation of miR-34a sensitizes bladder cancer cells to chemotherapy

**DOI:** 10.1186/1476-4598-13-8

**Published:** 2014-01-15

**Authors:** Heng Li, Gan Yu, Runlin Shi, Bin Lang, Xianguo Chen, Ding Xia, Haibing Xiao, Xiaolin Guo, Wei Guan, Zhangqun Ye, Wei Xiao, Hua Xu

**Affiliations:** 1 Department of Urology, Tongji Hospital, Tongji Medical College, Huazhong University of Science and Technology, Wuhan 430030, China; 2 Translational Medicin Center, Tongji Hospital, Tongji Medical College, Huazhong University of Science and Technology, Wuhan 430030, China; 3 School of Health Sciences, Macao Polytechnic Institute, Macao, China; 4 Department of Urology, First Affiliated Hospital of Anhui Medical University, Hefei, Anhui 230022, China

**Keywords:** miR-34a, Muscle invasive bladder cancer, Promoter hypermethylation, Chemosensitivity, CD44

## Abstract

**Background:**

Accumulating evidence suggests a tumor suppressive role for miR-34a in human carcinogenesis. However, its precise biological role remains largely elusive. This study aimed to reveal the association of the miR-34a expression and its modulation of sensitivity to cisplatin in muscle-invasive bladder cancer (MIBC).

**Methods:**

miR-34a expression in MIBC cell lines and patient tissues was investigated using qPCR. The methylation analysis of miR-34a promoter region was performed by MassARRAY. Synthetic short single or double stranded RNA oligonucleotides and lentiviral vector were used to regulate miR-34a expression in MIBC cells to investigate its function *in vitro* and *in vivo*.

**Results:**

miR-34a expression was frequently decreased in MIBC tissues and cell lines through promoter hypermethylation while it was epigenetically increased in MIBC cells following cisplatin treatment. Increased miR-34a expression significantly sensitized MIBC cells to cisplatin and inhibited the tumorigenicity and proliferation of cancer cells *in vitro* and *in vivo.* Furthermore, we identified CD44 as being targeted by miR-34a in MIBC cells following cisplatin treatment, and increased CD44 expression could efficiently reverse the effect of miR-34a on MIBC cell proliferation, colongenic potential and chemosensitivity.

**Conclusions:**

Cisplatin-based chemotherapy induced demethylation of miR-34a promoter and increased miR-34a expression, which in turn sensitized MIBC cells to cisplatin and decreased the tumorigenicity and proliferation of cancer cells that by reducing the production of CD44.

## Introduction

Bladder cancer is the ninth most common cancer diagnosis worldwide, with more than 330,000 new cases each year and more than 130,000 deaths per year [1]. At any point in time, 2.7 million people have a history of urinary bladder cancer [1]. At the initial diagnosis of bladder cancer, 70% of cases are diagnosed as non-muscle-invasive bladder cancer (NMIBC) and approximately 30% as muscle-invasive bladder cancer (MIBC). The standard treatment for patients with muscle-invasive bladder cancer is radical cystectomy. However, as approximately one-third of patients diagnosed with MIBC have undetected metastases at the time of treatment for the primary tumor and 25% of patients who undergo radical cystectomy present with lymph node involvement at the time of surgery, this ‘gold standard’ only provides 5-year survival in about 50% of patients [2]. In order to improve these unsatisfactory results, peri-operative chemotherapy has been explored since the 1980s. The updated analysis shows that cisplatin-based neoadjuvant chemotherapy significantly improves overall survival. However, as only about 50% of patients with MIBC will respond to cisplatin-based chemotherapy [3], there is still an urgent need to further investigate the mechanisms that prevent response to chemotherapy.

MiRNAs are endogenous, non-coding RNA molecules of approximately 19–25 nucleotides in length. Most miRNAs represses mRNA translation by blocking of translation, less frequently mRNA degradation, while a minor proportion of the miRNAs mediate mRNA target up-regulation [4]. Depending on their target genes, miRNAs have been shown to be associated with many types of cancers and involved in every aspect of cancer including proliferation, differation, metastasis and chemosensitivity [5]. Among these miRNAs, miR-34a has been described as a “star” miRNAs in cancer research, which commonly functions as a tumor suppressor and is down-regulated in many human cancers [6]. Furthermore, the aberrant miR-34a expression has been linked to chemotherapy resistance in a variety of cancer [7-11]. One relevant explanation for this is its regulatory function in p53 signaling pathway. miR-34a is the most significantly inducted miRNAs by p53 and ectopic miR-34a expression induces apoptosis, cell-cycle arrest, senescence and alters cancer cell chemosensitivity through direct targeting multiple genes in p53 signaling pathway, such as Sirt-1, CDK6, E2F3 and Bcl-2 [12-14]. However, this mechanism seems not fitting well in cisplatin-based bladder cancer chemotherapy as miR-34a chemosensitizes bladder cancer cells to cisplatin treatment regardless of p53-Rb pathway status [9]. So the regulatory mechanism of miR-34a in cisplatin-based bladder cancer chemotherapy is not clear and needs further investigation.

## Results

### miR-34a is frequently decreased in human MIBC tissues and cell lines through promoter hypermethylation

**Figure 1:**
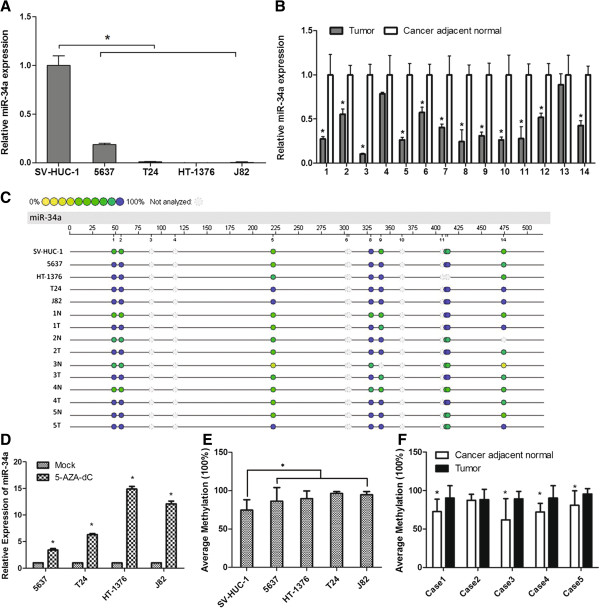
**MiR-34a was epigenetically downregulated in MIBC.** miR-34a expression was evaluated by qPCR in MIBC **A)** cell lines and **B)** patient tissues, U6 served as an internal control. Methylation analysis of miR-34a promoter region: **C)** profiling of the unit-specific methylation of CpG sites in the miR-34a promoter region was presented as an epigram. White circle means missing data at a given CpG unit; **D)** qRT-PCR demonstrated miR-34a expression in four MIBC cell lines after treatment with 5-aza-dC compared to mock-treated cells; Average methylation level of miR-34a promoter region in MIBC **E)** cell lines and **F)** patient tissues. Results are presented as mean + SEM of 3 independent experiments. *p < 0.05.

To investigate the role of miR-34a in MIBC, we firstly evaluated the expression of miR-34a in four MIBC cell lines (5637, HT1376, J82 and T24) and a non-tumorigenic bladder cell line SV-HUC-1 by qPCR. Compared to SV-HUC-1 cells, all of four MIBC cell lines had a significantly lower level of miR-34a expression (Figure 1A, P < 0.05). The expression of miR-34a was further analyzed by qPCR in 14 paired MIBC/adjacent normal bladder tissue specimens. And a highly significant (Figure 1B, P < 0.01) reduction of miR-34a in 12 of 14 MIBC tissues was observed.

To explore whether promoter hypermethylation leads to the suppression of expression, we examined the expression of miR-34a in bladder epithelial cell lines treated with the DNA methylation inhibitor, 5-aza-dC. After treatment, all the five cell lines showed a reactivation of miR-34a expression (Figure 1C). To further detect the promoter methylation status of the miR-34a quantitatively, the promoter CpG islands in bladder epithelial cell lines and tissue was determined by quantitative sequencing. As shown in Figure 1D, MassARRAY results showed hypermethylation was detected in all bladder cancer cell lines, while partial methylation in non-tumorigenic cell line SV-HUC-1, which was consistent with the 5-aza-dC detection. For tissues samples, 4 of 5 MIBC tissues showed hypermethylation while their paired cancer adjacent normal tissues showed partial methylation or unmethylation, which was consistent with the mRNA analysis (Figure 1B). Together, these results suggested that miR-34a was down-regulated through promoter hypermethylation in MIBC.

### Cisplatin can upregulate miR-34a by promoter demethylation in MIBC cell lines

**Figure 2:**
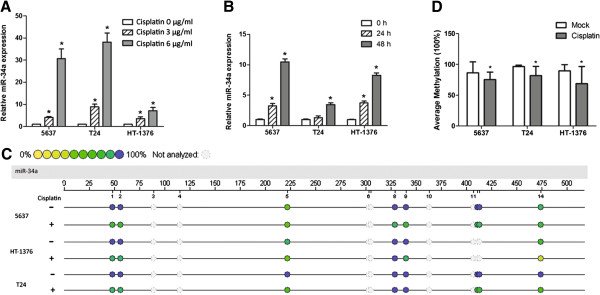
**Expression of miR-34a increased in MIBC cell lines via promoter demethylation following cisplatin treatment.** Cisplatin induced miR-34a expression in **A)** doze- and **B)** time-dependent manner, relative to control group ( cisplatin 0 μg/ml group or 0 h group, mean + SEM; n = 3; *p < 0.05). Methylation analysis of miR-34a promoter region: **C)** profiling of the unit-specific methylation of CpG sites in the miR-34a promoter region was presented as an epigram; **D)** Average methylation level of miR-34a promoter region in MIBC cell lines (mean + SEM; n = 3; *p < 0.05).

It is of note that aberrant expression of microRNA has been linked to chemosensitivity, so we next examined the expression of miR-34a in MIBC cells after cisplatin treatment. Result showed that chemotherapy led to increased miR-34a expression in all three MIBC cell lines in a dose- and time-dependent manner (Figure 2A and B). Subsequent epigenetic assessment also proved that cisplatin treatment induced miR-34a promoter demethylation (Figure 2C and D). Taken together, these findings provide evidence that cisplatin induced promoter demethylation could be an important reason for increased miR-34a expression in MIBC cell lines.

### Increased miR-34a expression sensitizes MIBC cells to cisplatin in vivo and in vitro

**Figure 3:**
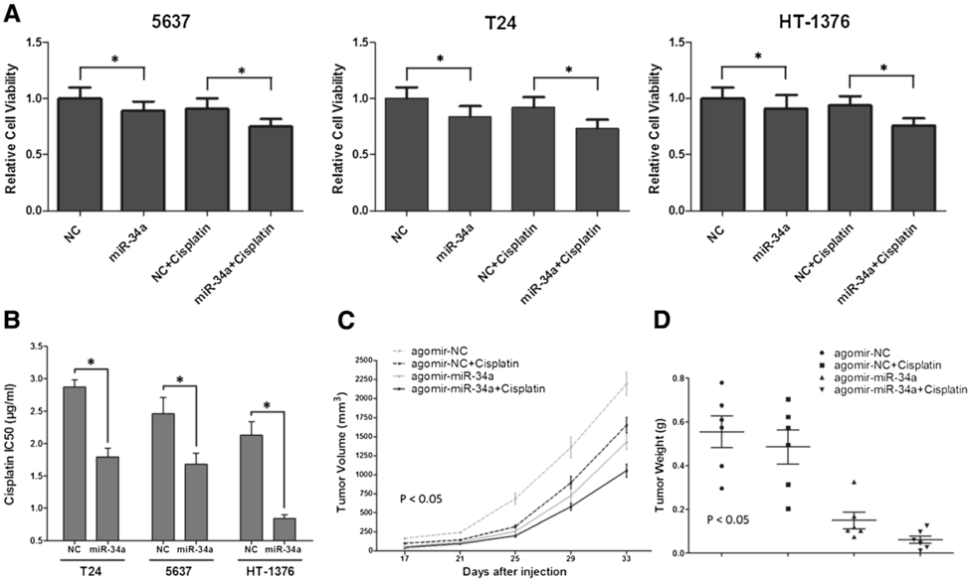
**Overexpression of miR-34a in MIBC cells sensitized tumor cells to cisplatin chemotherapy. A)** Tumor cell viability was detected by CCK-8 assay after different treatment. Data are plotted as the mean ± SEM of 3 independent experiments relative to mock treatments; **B)** The IC_50_ values for cisplatin of MIBC cell lines after transfected with miR-34a mimics (mean + SEM; n = 3; *p < 0.05); Mean xenograft tumor volume **C)** and weight **D)** in nude mice groups after indicated treatment (mean ± SEM; n = 3;).

As miR-34a expression increased dramatically following cisplatin treatment, we subsequently assessed in the association between miR-34a and chemosensitivity. Results of cell viability assay clearly showed that overexpression of miR-34a in 5637, T24 and HT1376 cells could efficiently increased their sensitivity to cisplatin as compared to NC group (Figure 3A and B, P < 0.05). This observation was further confirmed in T24 cell based xenograft model by using agomir-miRNA, a chemically modified miRNA oligonucleotide conjugated with cholesterol (Figure 3C and D). Importantly, statistical analysis demonstrated that the inhibition rate of each group satisfy the formula: (miR-34a + cisplatin) > (miR-34a alone) + (cisplatin alone), which indicated the synergistic effect of miR-34a to cisplatin chemotherapy.

### Elevated miR-34a expression inhibits proliferation and decreases clonogenic potential of MIBC cells in vivo and in vitro

**Figure 4:**
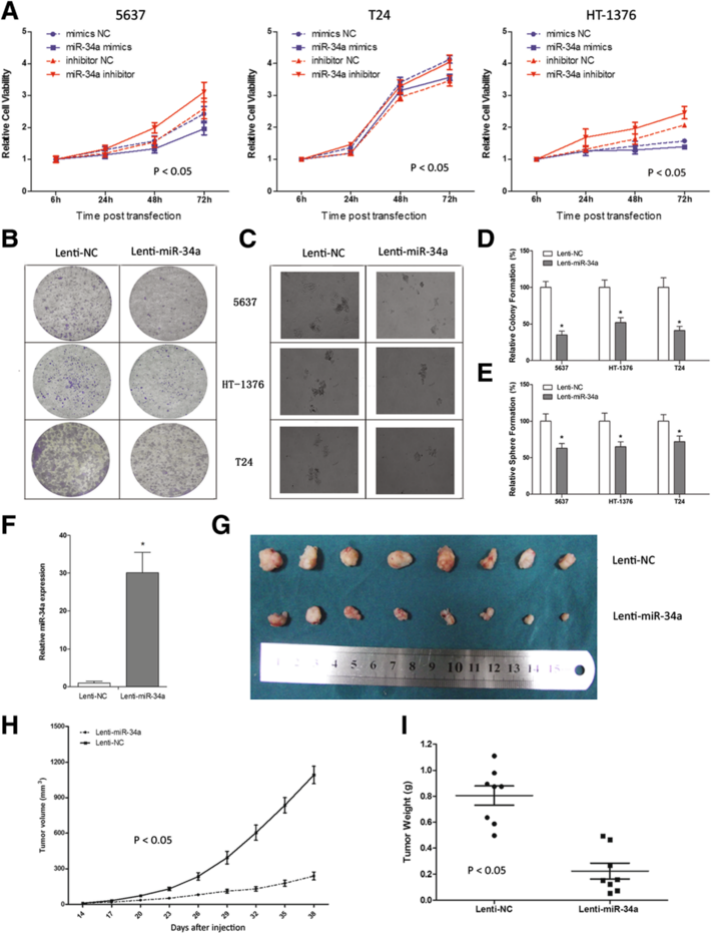
**MiR-34a functioned as a tumor suppressor in MIBC cells. A)** The effect of ectopic miR-34a expression on MIBC cell proliferation was investigated by CCK-8. The miR-34a activity was mediated by transfection with miR-34a mimics or inhibitor respectively. Data are plotted as the mean ± SEM of 3 independent experiments relative to mock treatments. The effect of ectopic miR-34a expression on MIBC cell tumorgenesis was investigated by **B)** colony-formation and **C)** sphere-formation assay. Quantitative analyses of **D)** colony and **E)** sphere numbers (mean + SEM; n = 3; *p < 0.05). **F)** Relative miR-34a expression in xenografts; **G)** Photographs of tumors excised 38 days after inoculation of stably transfected cells into nude mice; Mean xenograft tumor volume **H)** and weight **I)** in nude mice groups after indicated treatment (mean + SEM; n = 3; *p < 0.05).

In order to investigate the phenotypic consequences of miR-34a in MIBC cells, we overexpressed this miRNA in 5637, T24 and HT-1376 cells via miRNA mimics and Lentivectors. The proliferation and clonogenic potential were measured by CCK-8, colony formation and sphere formation assay respectively. And a synthetized miRNA inhibitor was also used in CCK-8 assay to further evaluate miR-34a function. Compared to NC group, overexpression of miR-34a significantly inhibited MIBC cell proliferation and vice versa (Figure 4A, P < 0.05), while as shown in Figure 4B-E, elevated miR-34a expression also dramatically decreased the colony and sphere formation ability of all three MIBC cell lines. Then T24 cells, which stably expressing miR-34a or NC, were used to construct the xenograft model in nude mice. As shown in Figure 4F, at d33 post injection, there were still significant miR-34a expression in derived xenograft. And when compared with NC group, miR-34a stably expressing tumor xenograft had markedly smaller size and lower weight (Figure 4G-I, P < 0.05). Taken together, these results demonstrated that miR-34a functions as a tumor suppressor in MIBC cells through decreasing their clonogenic potential.

### CD44 is a primary target of miR-34a in MIBC cells following cisplatin treatment

**Figure 5:**
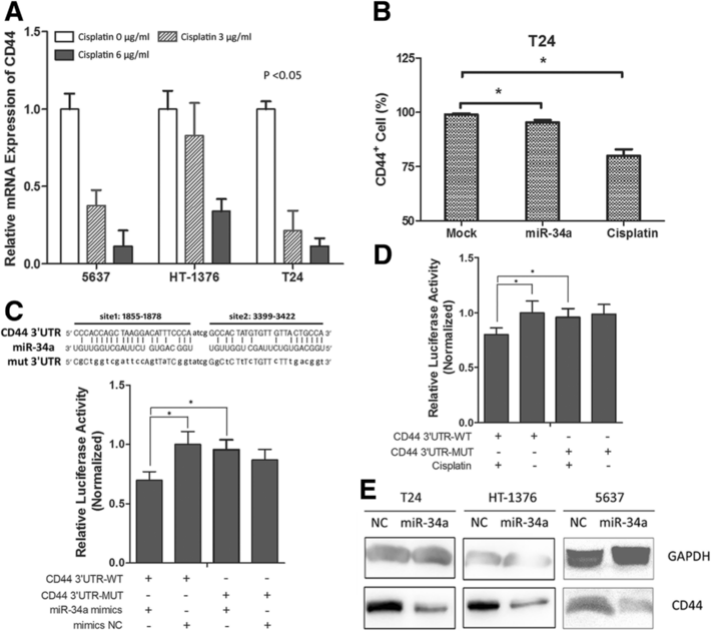
**CD44 was a primary target of miR-34a in MIBC cells following cisplatin treatment. A)** Relative CD44 expression in MIBC cells after cisplatin treatment relative to control group (cisplatin 0 μg/ml). **B)** CD44^+^ cells proportion decreased following miR-34a overexpression or cisplatin treatment. **C)** The seed regions of the miR-34a target sites in CD44 and the luciferase activity assay. **D)** Cisplatin-induced endogenous miR-34a upregulation caused the decrease of CD44-luciferase activity. **E)** CD44 protein expression was inhibited in miR-34a transfected MIBC cells. Data are plotted as the mean + SEM of 3 independent experiments. *p < 0.05.

To investigate the primary target of miR-34a that may help to explain the association between the increased miR-34a overexpression and cisplatin chemosensitivity, we initially measured the expression of some well-known targets of miR-34a in 5637, T24 and HT-1376 cells following cisplatin treatment, including MYC, TP53, BCL2, NOTCH1, CDK6, SIRT1, E2F1, CDK4, HGF, NOTCH2, SOX2 and CD44. However, it seems that only the expression of CD44 decreased in a doze-dependent manner following cisplatin treatment, which correlated with the expression of miR-34a (Figure 5A and Additional file 1). Moreover, FACS results showed that the CD44^+^ bladder cancer stem cells in T24 cells decreased either transfected with miR-34a or treated with cisplatin (Figure 5B). Then by luciferase reporter system and immunobloting, we determined CD44 was exactly a target of miR-34a in MIBC cells (Figure 5C and E). Subsequently, we designed another experiment to further explore this issue. As described, the CD44-luc-vector we used just contained the seed regions of the miR-34a potentially target sites in CD44 3′UTR. So its luciferase activity could only be affected by miR-34a. Based on this, we measured the luciferase activity in CD44-luc-transfected 5637 cells after cisplatin treatment. As shown in Figure 5D, luciferase activities decreased significantly in the CD44-luc-transfected 5637 cells when treated with cisplatin and such inhibition of luciferase activities by cisplatin could be abolished when the potential miR-34a binding sites were mutated. These results indicated that the downregulation of CD44 expression following cisplatin treatment was mainly due to cisplatin-induced endogenous miR-34a upregulation.

### Increased CD44 expression can efficiently reverse the effect of miR-34a on MIBC cell proliferation, colongenic potential and chemosensitivity

**Figure 6:**
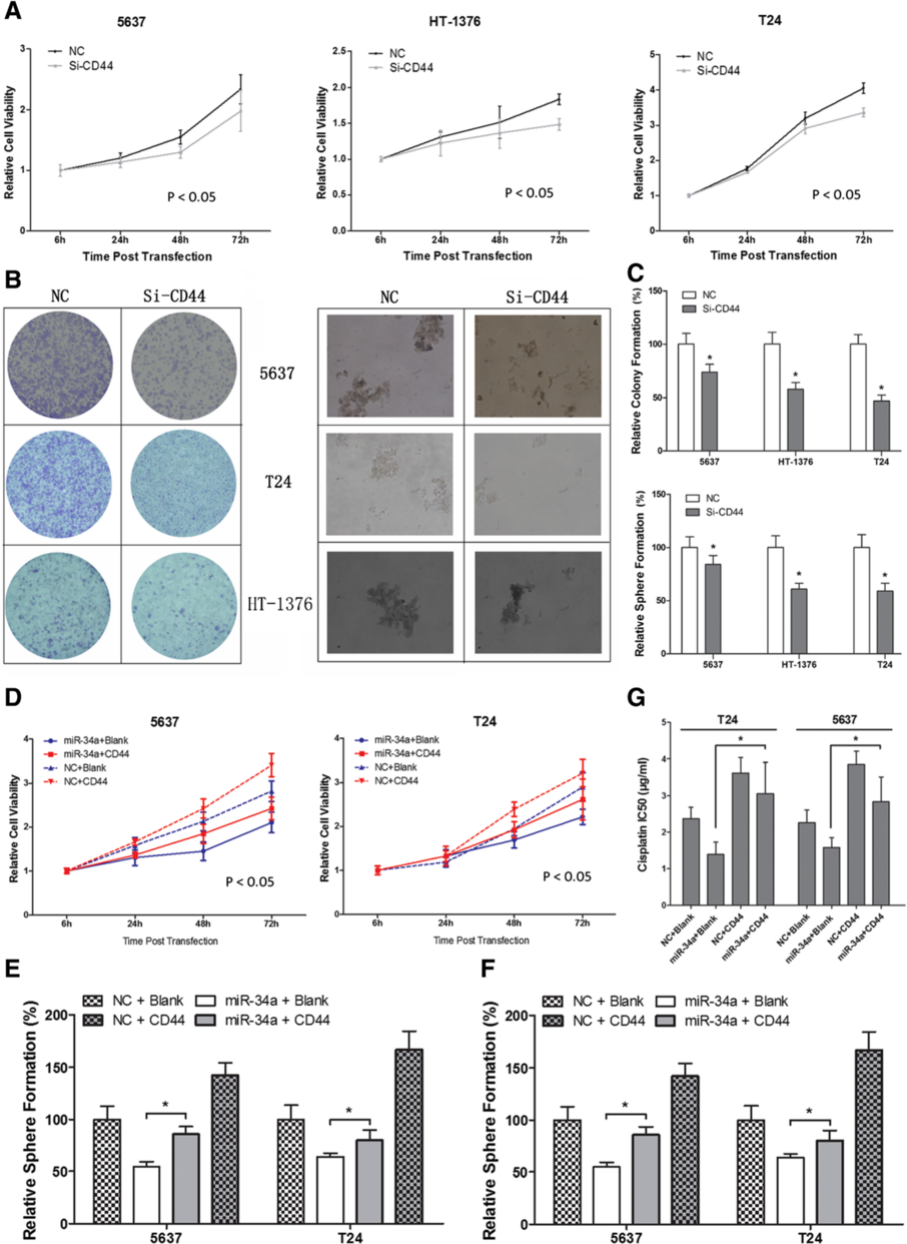
**The tumor-suppressive and chemosensitivity functions of miR-34a were mediated by reduction the production of CD44.** Downregulation of CD44 by siRNA led to similar effect of miR-34a overexpression on **A)** cell proliferation (mean ± SEM; n = 3; *p < 0.05) and **B-C)** tumorigenity (mean + SEM; n = 3; *p < 0.05). Increased CD44 expression could efficiently reverse the effect of miR-34a on MIBC **D)** cell proliferation (mean ± SEM; n = 3; *p < 0.05), **E-F)** colongenic potential and **G)** chemosensitivity (mean + SEM; n = 3; *p < 0.05).

We then tested whether miR-34a inhibited MIBC cell proliferation, colongenic potential and chemosensitivity through targeting CD44. Firstly, by using a CD44 siRNA, we demonstrated that downregulation of CD44 could efficiently inhibit cell proliferation (Figure 6A), decrease colony and sphere formation ability (Figure 6B and C) in all three MIBC cell lines, which correlated with the effect of overexpressed miR-34a. Subsequently, these assays were repeated when miR-34a mimics and CD44 expressing vector were co-transfected into 5637 and T24 cells. As shown in Figure 6D-F, CD44 overproduction appeared to have a dramatic positive effect on tumor cell growth and tumorigenesis, and importantly, miR-34a induced tumor suppression was largely eliminated upon the overexpression of CD44. Finally, we measured the IC_50_ values of cisplatin for these MIBC cell lines in differently treated group (Figure 6G), and the results showed that overexpression of CD44 could also efficiently reverse the effect of miR-34a on chemosensitivity. All these data presented here strongly suggesting that the tumor-suppressive and chemosensitivity effect of miR-34a was mediated by reducing the production of CD44 as its predominant target.

## Discussion

Bladder cancer is caused by a series of genetic alterations [15]. Recently, some miRNA expression profiling study pointed out that the aberrant expression of some miRNA had strong relationship with bladder cancer progression [16-19]. miR-34a was one of such miRNAs, which had been extensively studied in bladder cancer and many other cancers [8,10,20-24]. However, there was still a limit in studies focusing on miR-34a in MIBC. In this study, we determined that miR-34a expression was frequently decreased in MIBC cell lines and tumor tissues, which was similar in other cancers. Then, the possible mechanism that may help to explain the decrease of miR-34a in MIBC was investigated. Vogt et, al indicated that promoter hypermethylation could be an important reason for the decreased miR-34a expression in urothelial cancer [24], in our study, using a more exact method, Massaraay, which could detected up to 500 bp nucleotides containing 14 CpGs, combined with the data of 5-aza-dC demethylation treatment, we clearly demonstrated that promoter hypermethylation caused the decrease of miR-34a expression.

The chemosensitivity of MIBC cells was another area of our concern. Our present results indicated that there was a strong association between cisplatin treatment and miR-34a expression in MIBC. Moreover, we found the epigenetic alteration of miR-34a promoter following cisplatin treatment. It had been confirmed that cisplatin treatment upregulated miR-34a expression regardless of p53 status [9], while our results also indicated there was no significant correlation between miR-34a and P53 expression following cisplatin treatment (Figure 2A and Additional file 1), so the cisplatin induced epigenetic alteration could be a reasonable explanation for the upregulation of miR-34a expression. Subsequently, increased miR-34a expression induced by cisplatin could in turn improve cisplatin sensitivity of MIBC cells *in vitro* and *in vivo*. miR-34a has been described a tumor suppressive miRNA in many kinds of cancers through inducing tumor cell apoptosis, senescence, cell cycle arrest or repressing metastasis [9,10,14,20-22,25]. Our data presented here demonstrated that increased miR-34a expression could efficiently decrease tumorigenity of MIBC cells. This could partly explain its improved effect on chemosensitivity.

A miRNA’s function always relies on its targets. To investigate the primary target of miR-34a that could be the potential mechanism underlying the association between the increased miR-34a expression and cisplatin chemosensitivity, we initially measured the expression of some well-known targets of miR-34a in 5637, T24 and HT-1376 cells following cisplatin treatment, including MYC, BCL2, NOTCH1, CDK6, SIRT1, E2F1, CDK4, HGF, NOTCH2, SOX2 and CD44 [7,12,22,23,26,27]. Surprisingly, in spite of a glimpse through qRT-PCR on the level of mRNA, we still observed a clear inverse correlation between miR-34a and CD44 expression, but not others. Further experiments validated that the tumor-suppressive and chemosensitivity effect of miR-34a was mediated by reducing the production of CD44. As we know, CD44 have been described as a marker of human bladder cancer stem cells (CSCs), which have been reported to be resistant to therapeutics [28-30]. Many studies used chemotherapeutic treatment to enrich cancer stem cells. For example, the CD44^+^CD24^lo/2^ breast CSCs are enriched in breast cancer patients who have received adjuvant chemotherapy [31] and more resistant to some chemotherapeutic drugs [32]. In mouse models of mammary tumors, CSCs have also been shown to be refractory to cisplatin treatment [33]. Furthermore, chemoresistant colon cancer cells display CSC phenotypes [34] and CD133^+^ hepatic CSCs are chemoresistant due to preferential activation of the Akt pathway [35]. So here comes confusion. Base on speculations above, after cisplatin treatment, the bladder CSCs should be enriched and the CD44 expression and tumorigenity of the drug resistant cells should be increased, which is opposite of our observations. We noticed that similar results in prostate cancer cells were also reported [36]. These findings suggested that not all chemotherapeutic treatment is suitable to enrich cancer stem cells.

## Conclusion

In this study, we demonstrate that expression of miR-34a is frequently decreased in bladder cancer tissues and cell lines through promoter hypermethylation while it is epigenetically increased in bladder cancer cells following cisplatin treatment. Increased miR-34a expression significantly sensitizes bladder cancer cells to cisplatin treatment and inhibits the tumorigenicity and proliferation of cancer cells *in vitro* and *in vivo* through targeting CD44.

## Methods

### Patients and samples

In this study, 15 paired MIBC and adjacent normal tissue specimens were collected from patients who were diagnosed histopathologically with MIBC and received radical cystectomy between September 2010 and December 2011 in Tongji Hospital, Tongji Medical College, Huazhong University of Science and Technology. All the samples were immediately snap frozen and stored in liquid nitrogen. To obtain homogeneous and histological well-characterized samples for RNA analyses, the nature of the tissue and its specified composition were determined by an experienced pathologist. The study was approved by the Institutional Review Board and Ethics Committee of Tongji Hospital, Tongji Medical College, Huazhong University of Science and Technology, and written informed consent was obtained from all patients.

### Cell lines and culture

MIBC cell lines 5637, T24, J82 and HT1376 were purchased from ATCC, 5637 and T24 were maintained in RPMI-1640 medium; J82 was maintained in DMEM medium; HT1376 was maintained in MEM medium; SV-HUC-1 was also purchased from ATCC and maintained in DMEM/F-12 medium. Cells were cultured supplemented with 10% fetal bovine serum (FBS) in a humidified atmosphere of 5% CO_2_ maintained at 37°C.

### Reagents and transfection

Six synthetic, chemically modified short single or double stranded RNA oligonucleotides (miR-34a mimics, mimics NC, miR-34a inhibitor, inhibitor NC, agomir-miR-34a and agomir-NC) were purchased from Ribo Biotech (Guangzhou, China). Agomir-miRNA is a chemically modified miRNA mimics conjugated with cholesterol. Prevalidated siRNA specific for CD44 and a nonsilencing siRNA control were purchased from Genepharma Biotech (Shanghai, China). Lenti-miR-34a and Lenti-NC were purchased from Genechem Biotech (Shanghai, China) Primary antibody CD44 (1:1000) and GAPDH (1:10000) were purchased from Sigma-Aldrich, St. Louis, MO. Oligonucleotide and plasmid transfection was done by using X-tremeGENE siRNA Transfection Reagent (Roche) and FuGene HD Transfection Reagent (Roche) respectively, according to the manufacture’s protocol. The lentivirus infection was carried out according to the manufacture’s protocol and he MOI for 5637, T24 and HT1376 cells was 10, 10 and 5 respectively.

### DNA extraction and methylation analysis

Genomic DNA was isolated using QIAamp DNA Mini Kit (Qiagen, Valencia, CA) and bisulfite modification of the genomic DNA was carried out using an Epitect Bisulfite Kit (Qiagen, Valencia, CA) according to the manufacturer’s instructions. Quantitative methylation analysis of the promoter of miR-34a was performed using the Sequenom MassARRAY platform (CapitalBio, Beijing, China) as previous study description [37]. The sequence and primer sets used were shown in Additional file 2. The spectra methylation ratios were generated by Epityper software version 1.0 (Sequenom, San Diego, CA).

### Animal experiment

Tumorigenicity in nude mice was determined as described previously [23,38]. To evaluate the chemosensitivity effect of miR-34a, four groups of 6 mice each were injected subcutaneously with T24 cells at a single site. 17 days after injection when appreciable tumor formed subcutaneously, agomir-miR-34a or agomir-NC were injected combined with cisplatin or PBS in tumor. To evaluate the tumorigenity of miR-34a, two groups of 8 mice each were injected subcutaneously with miR-34a/NC stably expressing T24 cells constructed by lentivector infection and puromycin screening. Tumor onset measured with calipers at the site of injection every three day by two trained laboratory staffs at different times on the same day 14 weeks after injection when appreciable tumor formed subcutaneously. Tumor volume was calculated using the formula, V = 0.5ab^2^, where a represent the larger and b represents the smaller of the 2 perpendicular indexes. Animals were sacrificed 33 days or 38 days after injection and these tumors were weighed. Nude mice were manipulated and cared for in the Experiment Animal Center of the Tongji Medical College and was approved by the Ethics Committee of Tongji Medical College, Huazhong University of Science and Technology (Wuhan, Hubei, Province, P.R. China).

### Statistical analysis

At least three independent experiments were completed for each analysis described in this article. Data are shown as mean ± standard deviation (SD). Paired analysis was performed by Student’s t-test, and multiple group comparison was performed by one-way analysis of variance (ANOVA) using SPSS 19.0 software. P < 0.05 was considered statistically significant.

Additional methods are listed in Additional file 3: Supplementary Materials and Methods.

## Competing interests

The authors have declared no conflicts of interest.

## Authors’ contributions

HL carried out the epigenetic studies and drafted the manuscript. GY carried out the cell biology studies and participated in the animal experiments. HBX and RLS carried out the immunoassays and participated in the cell biology studies. DX and WG carried out all the qPCR assays. XLG participated in the animal experiments. BL and XGC performed the statistical analysis. ZQY acquised of funding and critically revised the manuscript for important intellectual content. W.X participated in its design and coordination and helped to draft the manuscript. HX conceived of the study, participated in its design and acquised of funding. All authors read and approved the final manuscript.

## Supplementary Material

Additional file 1 **Expression of some well-known targets of miR-34a in 5637, T24 and HT-1376 cells following cisplatin treatment.** mRNA expression of indicated genes were detected by qPCR. Click here for file

Additional file 2 CpG sites in the promoter region of miR-34a (n=14), and sequences of the primers used for amplification of converted DNA for sequenom massarray analysis. Click here for file

Additional file 3 Supplementary materials and methods. Click here for file
