# Erratum to: Cisplatin-induced epigenetic activation of miR-34a sensitizes bladder cancer cells to chemotherapy

**DOI:** 10.1186/1476-4598-13-183

**Published:** 2014-08-14

**Authors:** Heng Li, Gan Yu, Runlin Shi, Bin Lang, Xianguo Chen, Ding Xia, Haibing Xiao, Xiaolin Guo, Wei Guan, Zhangqun Ye, Wei Xiao, Hua Xu

**Affiliations:** Department of Urology, Tongji Hospital, Tongji Medical College, Huazhong University of Science and Technology, Wuhan, 430030 China; Translational Medicin Center, Tongji Hospital, Tongji Medical College, Huazhong University of Science and Technology, Wuhan, 430030 China; School of Health Sciences, Macao Polytechnic Institute, Macao, China; Department of Urology, First Affiliated Hospital of Anhui Medical University, Hefei, Anhui 230022 China

## Correction

After the publication of this work [1] it was brought to the authors’ attention that Figures six (Figure 1 here) (E) and (F) contained an error in their data presentation. The correct figure is given below.

**Figure 1:**
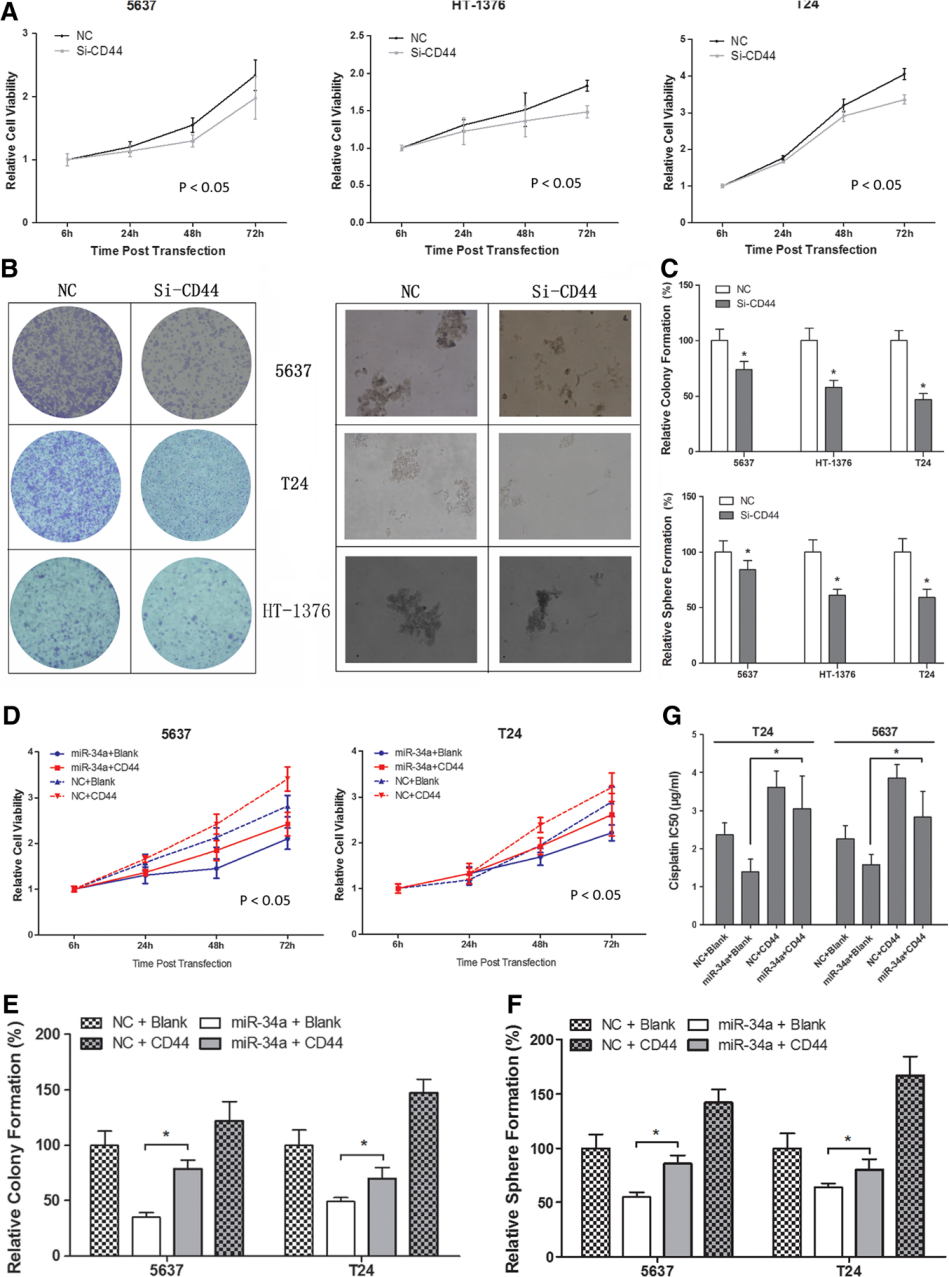
**The tumor-suppressive and chemosensitivity functions of miR-34a were mediated by reduction the production of CD44.** Downregulation of CD44 by siRNA led to similar effect of miR-34a overexpression on **A)** cell proliferation (mean ± SEM; n = 3; *p < 0.05) and **B-C)** tumorigenity (mean + SEM; n = 3; *p < 0.05). Increased CD44 expression could efficiently reverse the effect of miR-34a on MIBC **D)** cell proliferation (mean ± SEM; n = 3; *p < 0.05), **E-F)** colongenic potential and **G)** chemosensitivity (mean + SEM; n = 3; *p < 0.05).

We regret any inconvenience that this inaccuracy may have caused.
