# Correction: Cisplatin-induced epigenetic activation of miR-34a sensitizes bladder cancer cells to chemotherapy

**DOI:** 10.1186/s12943-022-01614-9

**Published:** 2022-07-28

**Authors:** Heng Li, Gan Yu, Runlin Shi, Bin Lang, Xianguo Chen, Ding Xia, Haibing Xiao, Xiaolin Guo, Wei Guan, Zhangqun Ye, Wei Xiao, Hua Xu

**Affiliations:** 1 Department of Urology, Tongji Hospital, Tongji Medical College, Huazhong University of Science and Technology, Wuhan, 430030 China; 2 Translational Medicin Center, Tongji Hospital, Tongji Medical College, Huazhong University of Science and Technology, Wuhan, 430030 China; 3 School of Health Sciences, Macao Polytechnic Institute, Macao, China; 4 Department of Urology, First Affiliated Hospital of Anhui Medical University, Hefei, 230022 Anhui China

**Correction: Mol Cancer 13, 8 (2014)**

**https://doi.org/10.1186/1476-4598-13-8**

Following publication of the original article [1], an error was identified in the images presented in Fig. 4, specifically:

**Fig. 4:**
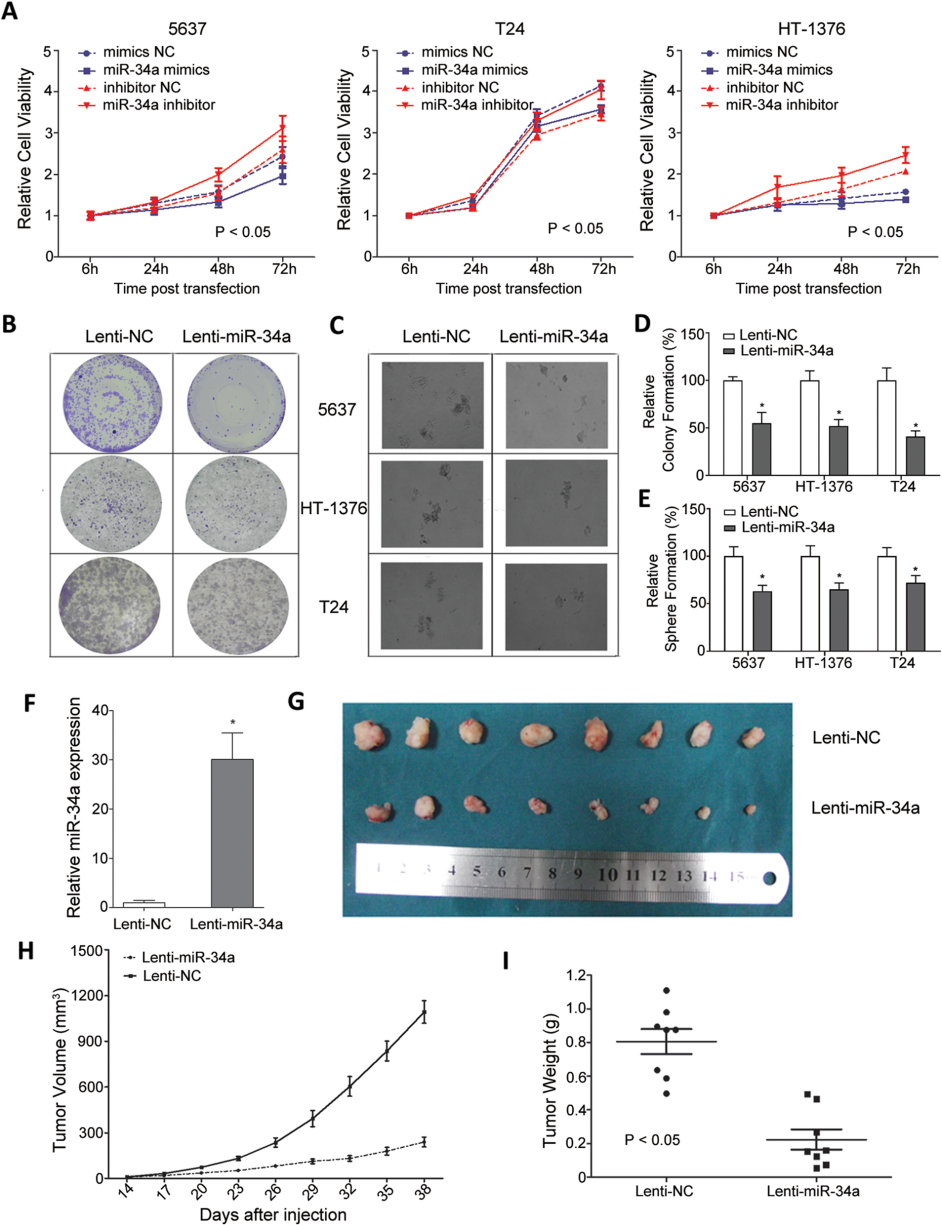
MiR-34a functioned as a tumor suppressor in MIBC cells. **A** The effect of ectopic miR-34a expression on MIBC cell proliferation was investigated by CCK-8. The miR-34a activity was mediated by transfection with miR-34a mimics or inhibitor respectively. Data are plotted as the mean ± SEM of 3 independent experiments relative to mock treatments. The effect of ectopic miR-34a expression on MIBC cell tumorgenesis was investigated by **B**) colony-formation and **C**) sphere-formation assay. Quantitative analyses of **D**) colony and **E**) sphere numbers (mean + SEM; *n* = 3; *p < 0.05). **F** Relative miR-34a expression in xenografts; **G**) Photographs of tumors excised 38 days after inoculation of stably transfected cells into nude mice; Mean xenograft tumor volume H) and weight **I**) in nude mice groups after indicated treatment (mean + SEM; *n* = 3; *p < 0.05)

Figure 4B: incorrect images were originally used for Lenti-NC in 5637 cell line; the correct images are now used

Furthermore, two of the primer sequences listed in the Table S1 (part of ‘Additional file 3: Supplementary materials and methods’) contained typographical errors. The sequences for HGF F and GAPDH F have been corrected in the new version of the file.

The authors provided the journal with the original data files. The corrected version of Fig. 4 is provided here. The correction does not have any effect on the results or conclusions of the paper.

## Supplementary Information

**Additional file 3: Table S1**. Primer sets sequences used for qPCR in this study. **Figure S1**. CpG sites in the promoter region of miR-34a (n=14), and sequences of the primers used for amplification of converted DNA for sequenom massarray analysis. **Figure S2**. Expression of some well-known targets of miR-34a in 5637, T24 and HT-1376 cells following cisplatin treatment. mRNA expression of indicated genes were detected by qPCR.
