# Supplementary figures and images for: Cisplatin-induced epigenetic activation of miR-34a sensitizes bladder cancer cells to chemotherapy
Source: Mol Cancer. 2014 Jan 15;13:8. doi: 10.1186/1476-4598-13-8 (PMC4022035; doi:10.1186/1476-4598-13-8)

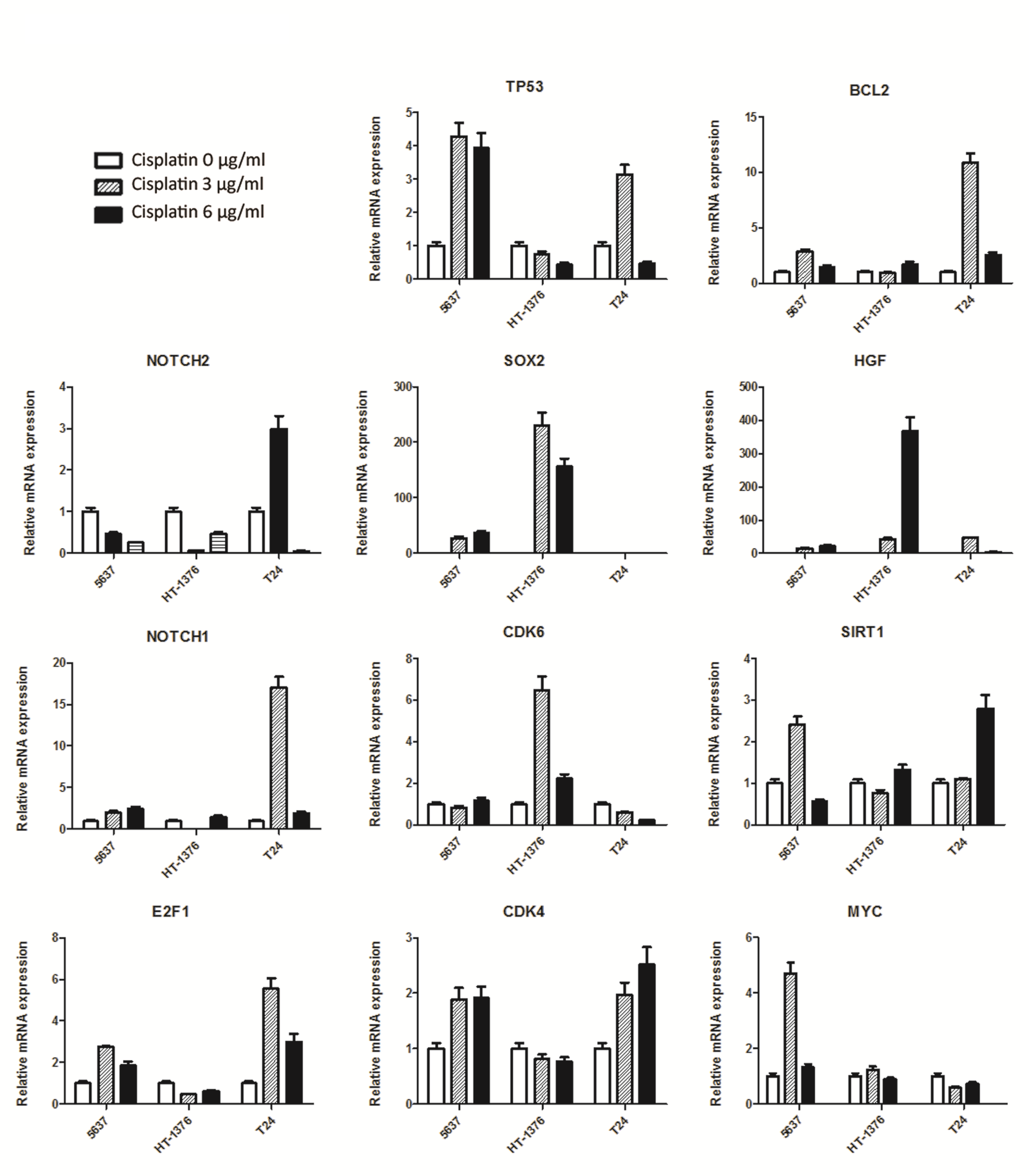

Supplement: Additional file 1 — Expression of some well-known targets of miR-34a in 5637, T24 and HT-1376 cells following cisplatin treatment. mRNA expression of indicated genes were detected by qPCR. [file 1476-4598-13-8-S1.tiff]

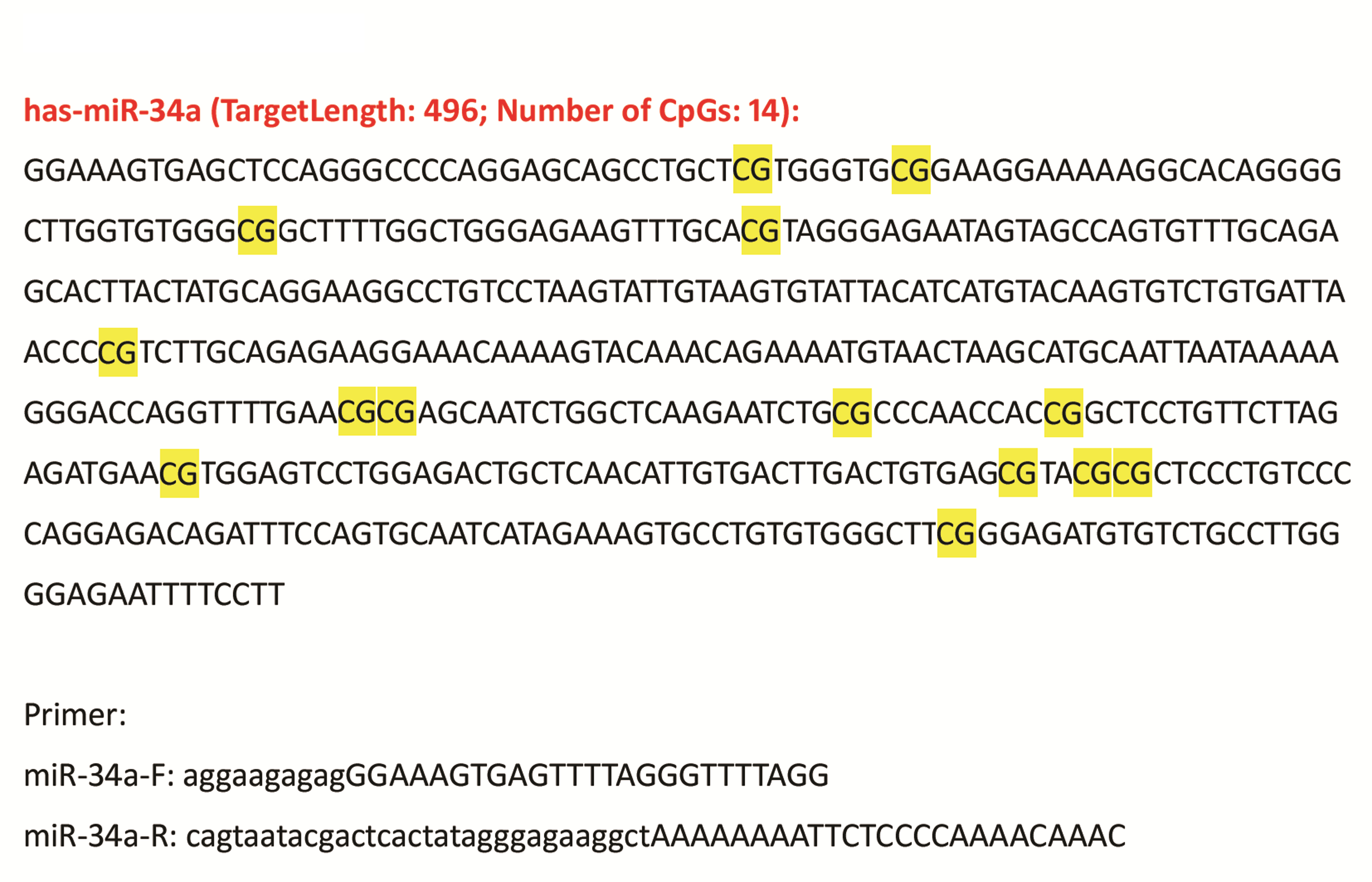

Supplement: Additional file 2 — CpG sites in the promoter region of miR-34a (n=14), and sequences of the primers used for amplification of converted DNA for sequenom massarray analysis. [file 1476-4598-13-8-S2.tiff]
