# Supplementary material for: Correction: Cisplatin-induced epigenetic activation of miR-34a sensitizes bladder cancer cells to chemotherapy
Source: Mol Cancer. 2022 Jul 28;21:155. doi: 10.1186/s12943-022-01614-9 (PMC9336090; doi:10.1186/s12943-022-01614-9)
Supplement: Supplementary file 1 — Additional file 3: Table S1. Primer sets sequences used for qPCR in this study. Figure S1. CpG sites in the promoter region of miR-34a (n=14), and sequences of the primers used for amplification of converted DNA for sequenom massarray analysis. Figure S2. Expression of some well-known targets of miR-34a in 5637, T24 and HT-1376 cells following cisplatin treatment. mRNA expression of indicated genes were detected by qPCR. [file 12943_2022_1614_MOESM1_ESM.rtf]

Supplementary Materials and Methords
RNA extraction and qRT-PCR
RNA was isolated using Qiagen miRNeasy kits (Qiagen, Valencia, CA). RNA concentration and purity was assessed using a Nanodrop 1000 spectrophotometer. MiR-34a and U6 expression was assessed using predesigned miR-34a and U6 primer sets (Ribo Biotech, Guangzhou, China) in combination with the Fermentas RT reagent Kit (Thermo Scientific, USA) and Universal SYBR PCR Master Mix kits (TAKARA, Dalian, China) according to the manufacturer's instructions. Primers used for Myc, Bcl-2, Notch1, CDK6, SIRT1, E2F1, CDK4, HGF, Notch2, SOX2, TP53 and CD44 mRNA expression detection were listed in Supplementary Table 1.
Cell proliferation assay
MIBC cells were transfected with RNA oligonucleotides for approximately 6 hours. Following treatments, cells were transferred to 96 well micro-plates and seeded at a density of approximately (5637: 1500 cells/well), (T24: 1000 cells/well), (HT-1376: 2000cells/well). Then cell viability was subsequently determined every 24 hours for 3 days by using the Cell Counting Kit-8 (CCK-8, Dojindo, Japan) according to the manufacturer's protocol with a micro-plate reader (MULTISCAN MK3, Thermo Scientific, USA) at the absorbance of 450nm.
Colony and Sphere formation assay
For colony formation assay, cells were treated and placed in 6-well plate at a density of 1000 cells/well and maintained in media containing 10% FBS for 10-12 days. Culture medium was changed every 3 days. Colonies were fixed with methanol and stained with 0.1% crystal violet in 20% methanol for 15 minutes. For sphere formation assay, 500 cells were placed in each well of 6-well plate and maintained in media containing 10% FBS for 10 days with any movement or treatment. The number of colonies or spheres was counted using an inverted microscope (Olympus, Japan).
Luciferase reporter assay
A 72bp DNA sequence containing the two miR-34a binding sites (see Fig. 5B) in CD44 3'UTR and the corresponding mutant constructs were synthetized and cloned into psiCHECK-2 Luciferase vector. The constructs were veriﬁed by sequencing. For luciferase reporter assays, 5637 cells were seeded in 24-well plates and transiently co-transfected with appropriate reporter plasmid and miRNA. After 48h, the cells were harvested and lysed, and luciferase activity was measured using the Dual-Luciferase Reporter Assay System (Promega, Madison, WI, USA) in a multi-function micro-plate reader (Tristar2 LB942, Berthold, Germany). Firefly-luciferase was used for normalization.
Supplementary Table
Table S1. Primer sets sequences used for qPCR in this study.

Gene	Primer Sequence (5'-3')	
Myc	F: GGCTCCTGGCAAAAGGTCA	
	R: CTGCGTAGTTGTGCTGATGT	
P53	F: CAGCACATGACGGAGGTTGT	
	R: TCATCCAAATACTCCACACGC	
Bcl-2	F: GGTGGGGTCATGTGTGTGG	
	R: CGGTTCAGGTACTCAGTCATCC	
Notch-1	F: GAGGCGTGGCAGACTATGC	
	R: CTTGTACTCCGTCAGCGTGA	
Notch-2	F: CAACCGCAATGGAGGCTATG	
	R: GCGAAGGCACAATCATCAATGTT	
Cdk6	F: GCTGACCAGCAGTACGAATG	
	R: GCACACATCAAACAACCTGACC	
Sirt-1	F: TAGCCTTGTCAGATAAGGAAGGA	
	R: ACAGCTTCACAGTCAACTTTGT	
E2F1	F: ACGCTATGAGACCTCACTGAA	
	R: TCCTGGGTCAACCCCTCAAG	
Cdk4	F: ATGGCTACCTCTCGATATGAGC	
	R: CATTGGGGACTCTCACACTCT	
HGF	F: TCCATGATACCACACGAACACA	
	R: CGTAGCGTACCTCTGGATTGC	
CD44	F: TCCAACACCTCCCAGTATGACA	
	R: GGCAGGTCTGTGACTGATGTACA	
GAPDH
	F: GCAGGCGTCGGAGGGCCCCCTC
R: GGGACTGAGTGTGGCAGGGACTCC	


Supplementary figure legends
Fig. S1 CpG sites in the promoter region of miR-34a (n=14), and sequences of the primers used for amplification of converted DNA for sequenom massarray analysis.

Fig. S2 Expression of some well-known targets of miR-34a in 5637, T24 and HT-1376 cells following cisplatin treatment. mRNA expression of indicated genes were detected by qPCR.
